# Supplementary material for: A workflow for the detection of antibiotic residues, measurement of water chemistry and preservation of hospital sink drain samples for metagenomic sequencing
Source: J Hosp Infect. Author manuscript; Available in PMC 2025 Mar 10. (PMC7617466; doi:10.1016/j.jhin.2023.11.021)
Supplement: Supplementary Figure 1 [file EMS203362-supplement-Supplementary_Figure_1.docx]

**
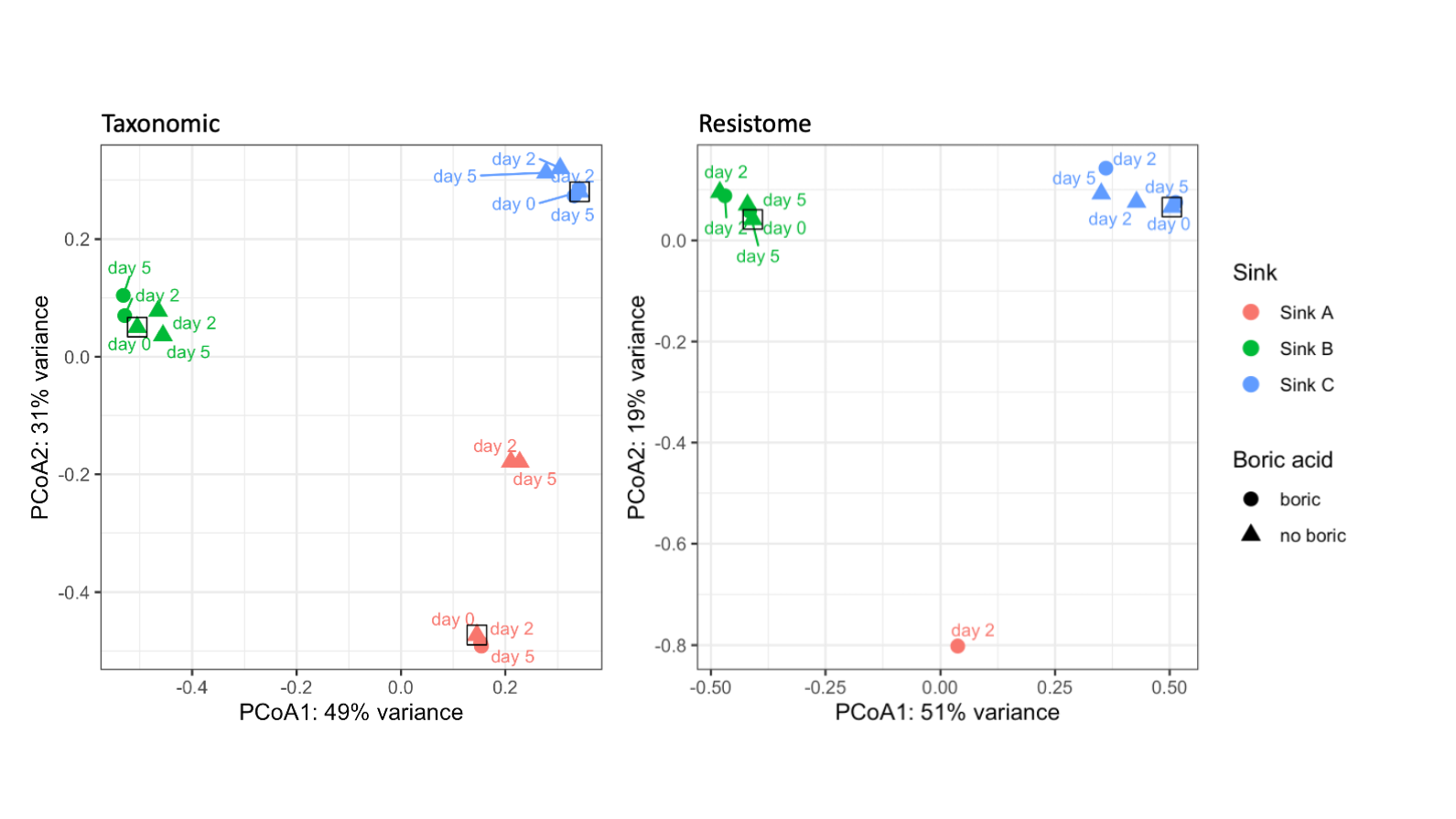
**

**Supplementary figure 1 Principal coordinate analyses of sample-level Bray-Curtis dissimilarities for (left panel) taxonomic and (right panel) resistome content.** Points represent individual samples with colour denoting sink and shape denoting the use of boric acid, with baseline samples highlighted (square outline). N.B. no AMR genes were detected for Sink A Day 0 or 5 +/- boric acid.
