# Supplementary material for: A workflow for the detection of antibiotic residues, measurement of water chemistry and preservation of hospital sink drain samples for metagenomic sequencing
Source: J Hosp Infect. Author manuscript; Available in PMC 2025 Mar 10. (PMC7617466; doi:10.1016/j.jhin.2023.11.021)
Supplement: Supplementary Table 1 [file EMS203362-supplement-Supplementary_Table_1.docx]

| **Beta-lactams** | **Tetracyclines** | **Sulfonamides** | **Quinolones** |
| --- | --- | --- | --- |
| Penicillin G | Tetracycline | Sulfamethizole | Enoxacin |
| Ampicillin | Oxytetracycline | Sulfamethoxazole | Sarafloxacin |
| Amoxicillin | Doxycycline | Sulfadiazine | Ofloxacin |
| Oxacillin | Chlortetracycline | Sulfadimidine | Ciprofloxacin |
| Cloxacillin |  | Sulfachloropyridine | Pefloxacin |
| Dicloxacillin |  | Sulfamonomethoxine | Fleroxacin |
| Nafcillin |  | Sulfaquinoxaline | Enrofloxacin |
| Benzathine benzylpenicillin |  | Sulfachlorpyridazine | Danofloxacin |
| Procaine penicillin |  | Sulfamethoxypyridazine | Flumequine |
| **Cephalosporins** |  |  | Difloxacin |
| Cefquinome |  |  | Norfloxacin |
| Cefacetrile |  |  | Marbofloxacin |
| Cefalonium |  |  | Lomefloxacin |
| Cefoperazone |  |  |  |
| Cephapirin |  |  |  |
| Cefazolin |  |  |  |
| Cefuroxime |  |  |  |
| Ceftiofur |  |  |  |

**Supplementary table 1. List of antibiotics in each class that are detected by the QuaTest BTSQ 4-in-1 (Beta/Tetra/Sulfa/Quino) rapid test kit.**
